# An examination of procrastination in a multi-ethnic population of adolescents from New Caledonia

**DOI:** 10.1186/s40359-022-01032-y

**Published:** 2023-01-02

**Authors:** Stéphane Frayon, Viren Swami, Guillaume Wattelez, Akila Nedjar-Guerre, Olivier Galy

**Affiliations:** 1 Interdisciplinary Laboratory for Research in Education, EA 7483, School of Education, University of New Caledonia, BP R4, Avenue James Cook, 98851 Nouméa Cedex, New Caledonia; 2 School of Psychology and Sport Science, Anglia Ruskin University, Cambridge, UK; 3 Centre for Psychological Medicine, Perdana University, Serdang, Malaysia

**Keywords:** Procrastination, Pacific, Oceania, Ethnicity, Sex differences, Adolescence, Ethnic identity

## Abstract

**Background:**

Although procrastination has been widely studied in adults, comparatively little work has focused on adolescent procrastination, especially in the Pacific region. As a contribution to knowledge and diversification of population sampling, therefore, we examined procrastination in a multi-ethnic sample of adolescents from New Caledonia. Specifically, we examined gender and ethnic differences in procrastination, as well as sociodemographic and ethnic identity predictors of procrastination.

**Methods:**

927 adolescents (474 boys, 453 girls; age *M* = 13.2 years) completed measures of procrastination and ethnic identity, and reported their ethnicity (Kanak vs. Polynesian vs. European). Sociodemographic data (sex, age, area of residence and socioeconomic status) were also collected.

**Results:**

An analysis of variance indicated significant ethnic (Kanak and Polynesian adolescents had higher procrastination than European adolescents) and sex differences (girls had higher procrastination than boys), but no significant interaction. Regression analysis showed that higher procrastination was significantly associated with sex, ethnicity, age, and the interaction between ethnicity and ethnic identity. Moderation analysis showed that ethnic identity moderated the relationship between ethnicity and procrastination, but only in Kanak adolescents.

**Conclusion:**

Relatively high levels of procrastination were observed in Kanak and Polynesian adolescents, and in girls. These findings, while preliminary, may have important implications for academic attainment in the New Caledonian context.

## Background

*Procrastination* is a widespread phenomenon that can be defined as “the voluntary delay of an intended and necessary and/or [personally] important activity, despite expecting potential negative consequences that outweigh the positive consequences of delay” [1]. Although individuals differ in the extent to which they procrastinate [2], cross-national studies have reported that large numbers of adults—between 15 and 30%—engage in problematic procrastination [2–4]. Moreover, the prevalence of procrastination may be even higher in some populations: for instance, studies have reported that up to 50% of university students engage in problematic procrastination [5]. Such findings are important because procrastination is associated with a range of negative outcomes, including impaired work and academic performance [6–8], poorer psychological well-being [9, 10], negative health behaviours [11–13], and poorer sleep quality [14].

Although much is now known about procrastination in adult and especially university samples, surprisingly little research has been conducted with adolescent populations [15]. This is particularly important given the negative association between age and procrastination [2], suggesting that individuals get better at dealing with procrastination as they age. Indeed, some research has suggested that the prevalence of procrastination may be especially high in adolescence [16–18], with about 80% of adolescents procrastinating for an hour or more a day [19]. In particular, adolescents tend to procrastinate on unpleasant tasks, such as academic writing and anxiety-inducing tasks [20, 21]. Moreover, the consequences of procrastination in adolescence may be similarly deleterious as it is in adulthood. For example, procrastination in adolescence has been associated with significantly lower self-esteem, poorer academic achievement, greater symptoms of depression, and poorer sleep quality [22–25].

The little research that is focused on adolescent procrastination is primarily concerned with predictive factors. For instance, an important body of work has shown that perceived parental control is significantly associated with greater adolescent procrastination, whereas perceived parental autonomy is significantly associated with lower procrastination [26]. Likewise, parental attachment styles have also been found to be associated with adolescent procrastination, with maternal and paternal alienation in particular associated with significantly higher levels of procrastination [27]. Other research among adolescents has focused on uncontrolled Internet and smartphone use, which mediates relationships between procrastination and psychological functioning (e.g., sleep quality, stress; [28–30]). Beyond psychological traits and behaviours, other relevant research has focused on demographic characteristics that may shape procrastination.

Thus, a small handful of studies have examined sex and cross-national differences in procrastination. In terms of the former, whereas the evidence among adults suggests that men are significantly more likely to procrastinate than women (for meta-analyses, see [31, 32]), findings among adolescent samples are more equivocal. In samples of adolescents from Canada and Singapore, for example, boys were found to have significantly higher levels of procrastination than girls [19]. In contrast, studies of adolescents from Turkey have reported no significant sex difference in levels of procrastination [33, 34]. In terms of cross-national differences, Klassen et al. [19] reported that Singaporean adolescents had significantly higher levels of procrastination than their Canadian peers, which may be ascribed to cross-cultural differences in time orientation and centrality [35]. It remains the case, however, that the vast majority of studies on (adult) procrastination have relied on samples from Western, educated, industrialised, rich, and democratic nations (for exceptions, see [36, 37]).

As a contribution to the literature on adolescent procrastination, the present study sought to examine sociodemographic predictors of procrastination in a hitherto under-researched population, namely adolescents from New Caledonia. Located in the Pacific Ocean, New Caledonia is a special collectivity of France and is divided into three provinces (North Province, South Province, and Loyalty Islands Province), with provincial populations varying in ethnic composition, socioeconomic status (SES), and the degree of urbanisation. The ethnic composition of New Caledonia is especially important: at the 2014 census [38], 39.1% of the population reported having Kanak ancestry (i.e., part of the Melanesian group, indigenous to New Caledonia), 27.2% identified as European, and 10.3% identified as Polynesian (Tahitian and Wallisian), with other groups making up the remainder. While the Kanak tend toward more traditional, collectivist, and Pacific lifestyles and norms, Europeans and other ethnic groups have adopted a more Westernised way of life [39, 40].

A primary objective of the present study, therefore, was to examine possible ethnic differences in adolescent procrastination. This is important, firstly, because we are not aware of any previous study that has considered within-nation ethnic differences in adolescent procrastination. Doing so may, therefore, point to a unique intra-national factor that impacts upon procrastination, although it is difficult to determine *a priori* how that will play out in the context of New Caledonia. Second, any relationship between ethnicity and procrastination may be complicated by *ethnic identity* [41]. For instance, one previous study with New Caledonian adolescents reported that more positive ethnic identity was significantly associated higher self-esteem across ethnic groups [42]. In the present case, therefore, we considered the extent to which ethnicity and ethnic identity both in isolation and interactively were associated with procrastination.

### The present study

The overall objective of the present study was to examine sociodemographic differences in, and factors associated, with procrastination in a sample of adolescents from New Caledonia. More specifically, we first considered the relationship between procrastination and participant sex, with the expectation that boys would show significantly higher levels of procrastination that girls [31]. Second, we examined the extent to which both ethnicity and ethnic identity—in isolation and also interactively—were associated with adolescent procrastination. Given the dearth of research here, we considered these analyses to be exploratory. Similarly, for exploratory purposes, we also included socioeconomic status and urbanicity of residence in our analyses, as these may pertinent in the New Caledonian context [43]. Finally, we also included participant age, with the expectation that of a negative association between levels of procrastination and age [16]. We acknowledge at the outset the largely exploratory and preliminary nature of our study design, but believe this is offset—to some extent at least—by the inclusion of an under-investigated sample.

## Methods

### Participants

The sample for this study consisted of 927 adolescents (474 boys, 453 girls) recruited from New Caledonia (Kanak *n* = 452, European *n* = 375, Polynesian *n* = 100). Participants ranged in age from 10.5 to 16.1 years (*M* = 13.2, *SD* = 1.2). Full descriptive information about the sample is reported in Table 1.

### Materials

#### Procrastination

To measure the tendency to procrastinate, we used the Procrastination Assessment Scale–Students (PASS; [44]). The original PASS consists of 12 items that were specifically designed to assess procrastination in French-speaking populations (sample item: “When I have work to do, I wait a long time before starting”). However, Osiurak and colleagues [44] also recommended the removal of one item (Item #1) to improve composite reliability. Participants in the present study were, therefore, asked to complete the 11-item version of the PASS, with all items rated on a 5-point scale ranging from 1 (*false*) to 5 (*true*). Although the PASS has been shown to have adequate internal consistency, acceptable test-retest reliability up to 11 weeks, and adequate construct validity in French-speaking populations [44], it has not been previously used in New Caledonian adolescents. We, therefore, computed a principal-axis exploratory factor analysis (EFA) with the total sample, using a quartimax rotation. Bartlett’s test of sphericity, χ^2^[55] = 2185.69, *p* < .001, and the Kaiser–Meyer–Olkin measure of sampling adequacy (0.86) indicated that the 11 PASS items had adequate common variance for factor analysis. The results of the EFA revealed three factors with λ > 1, but parallel analysis indicated that only one factor from the actual data had λ greater than the criterion λ generated from the random data (λ_1_ = 3.03 > 1.78, λ_2_ = 0.53 < 1.27, λ_3_ = 0.40 < 1.01). As such, we retained one factor, which explained 28% of the common variance. Two PASS items (Items #7 and 5, following the numbering convention from the original 12-item version) had poor item-factor loadings (0.06 and 0.01, respectively), so were discarded. It is likely that this reflects method issues given that Items #7 and 5 are both negatively worded items; alternatively, it may be that these items are less relevant for adolescent populations, given that the PASS was originally developed with university students. All remaining PASS items had adequate item-factor loadings (> 0.46). A final PASS score was, therefore, computed as the mean of the nine retained items (McDonald’s omega = 0.81, 95% CI 0.79, 0.83).

#### Ethnic identity

Participants completed the Multigroup Ethnic Identity Measure–Revised [45, 46]. The 6-item MEIM-R is an abbreviated version of the MEIM [47], both of which assess affiliation with one’s ethnic group, and the instrument can be used with adolescents as young as 10 years [48]. Items were rated on a 5-point scale ranging from 1 (*strongly disagree*) to 5 (*strongly agree*). In its parent form [45] and in a range of national groups [49], MEIM-R scores have been found to consist of two factors indexing exploration of, and commitment towards, one’s ethnic group. However, among New Caledonian adolescents, MEIM-R scores have been found to reduce to a single dimension consisting of all 6 items [42]. We, therefore, computed an overall score as the mean of all 6 items, with higher scores reflecting greater affiliation with one’s ethnic group. Frayon and colleagues [42] reported that the MEIM-R had adequate composite reliability. In the present study, McDonald’s omega scores was 0.74 (95% CI 0.70, 0.78).

#### Ethnicity

Ethnicity was self-reported by the adolescents from a single question (“What community do you feel you belong to?”) and categorised as recommended by the INSERM report on New Caledonia [50], with the exception that we did not allow participants to choose more than one ethnic group. Participants from other ethnic groups (*N* = 64) were not included for analysis in the present study because of the small subsample size.

#### Sociodemographics

Socioeconomic status (SES) and area of residence were determined as previously described by Frayon and colleagues [42]. Briefly, SES was indexed on the basis of the occupation of the household reference person (defined as the householder with the highest income) and three categories were retained: managerial and professional occupations (higher SES), intermediate occupations (intermediate SES), and routine and manual occupations (lower SES). Area of residence was determined using a European standard [51].

#### Procedures

Ethics approval for this project was obtained from the Ethics Committee of the University of New Caledonia and the project was conducted in accordance with the requirements of the Declaration of Helsinki. As part of a larger study, eight secondary public schools were randomly selected to obtain a representative repartition between rural and urban areas, which is, in New Caledonia, 63% and 37%, respectively. The selection criterion was school size (*n* > 150) to ensure sufficient data in a single field trip. One or two classes were then randomly selected in each of four grades (levels) by a staff member, for a total of approximatively 150 students (6 groups with a mean of 25 students per division). We obtained only 89.5% of the expected data due to parental refusal or no return of the parental agreement. Participants completed an anonymous, paper-and-pencil questionnaire consisting of the measures described above. Adolescents with missing data (*n* = 71; i.e., around 7%) for one value were then excluded from analyses. Parents gave informed written consent prior to the adolescents’ participation in the study and all participants received written debriefing information at the end of the study. Data were between July 2018 and April 2019.

### Statistical analysis

All analyses were conducted using IBM SPSS v.25. We first examined between-group (Kanak vs. European vs. Polynesian) differences in sample characteristics using univariate analyses of variance (ANOVAs) or χ^2^ tests. Next, we computed a 3 × 2 ANOVA with ethnicity (Kanak vs. European vs. Polynesian) and sex (boys and girls) entered as independent variables and PASS scores as the dependent variable. Multivariable linear regression were used to explored factors that were significantly associated with procrastination in each ethnic group separately, as previously described by Frayon and colleagues [42]. PASS scores were the criterion variable and predictor variables in the model were age, sex, SES, area of residence, and MEIM scores. Gender, area of residence, and SES were categorised into groups by creating dummy variables. The moderation effect of ethnic background on the relationship between ethnic identity and procrastination was determined using the PROCESS macro (release 3.4.1) [52]. Ethnicity was the moderator, procrastination was the dependent variable, and the predictor was ethnic identity. Other variables not used in the models (age, SES, area of residence, gender) were entered as covariates.

## Results

### Sample characteristics and preliminary analyses

Table 1 presents descriptive statistics as a function of ethnicity. Preliminary analyses indicated significant between-group differences in SES (European adolescents had significantly higher SES than Kanak and Polynesian adolescents) and area of residence (Kanak and Polynesian adolescents mostly lived in rural areas, while European and adolescents from other origin mostly lived in urban areas), and ethnic identity (Polynesian and Kanak adolescents had significantly higher scores than adolescents from European and other origin). There were no significant between-group differences in the distribution of sex and age.

**Table 1:** Sociodemographic characteristics and scores of the participants by ethnicity

		Kanak (*N* = 452)		European (*N* = 375)		Polynesian (*N* = 100)				
		*N*	%	*N*	%	*N*	%	χ^2^	*df*	*p*
*Sex*										
Boys		225	49.8	198	52.8	51	51.0	0.8	2	0.687
Girls		227	50.2	177	47.2	49	49.0			
*Socio-economic status*										
High		104	23.0	258	68.8	28	28.0	208.3	4	< 0.001
Intermediate		143	31.6	83	22.1	34	34.0			
Low		205	45.4	34	9.1	38	38.0			
*Area of residence*										
Urban		63	13.9	257	68.5	30	30.0	262.8	2	< 0.001
Rural		389	86.1	118	31.5	70	70.0			

		*M*	*SD*	*M*	*SD*	*M*	*SD*	F	*df*	
Age (years)		13.26	1.2	13.15	1.21	13.14	1.2	0.9	2	0.398
Ethnic identity		3.21	0.52	2.94	0.60	3.29	0.52	29.6	2	< 0.001

### Sex and ethnic differences in procrastination

Descriptive statistics for levels of procrastination as a function of sex and ethnicity are reported in Table 2. A 3 × 2 ANOVA showed no significant ethnicity by sex interaction, *F*(2, 927) = 0.09, *p* = .916. There was, however, a significant main effect of ethnicity, *F*(2, 927) = 20.67, *p* < .001, η_p_^2^ = 0.06. Tukey testing indicated that Kanak and Polynesian adolescents had significantly higher procrastination score than European adolescents (*p* < .001; *d*s = 0.47 and 0.70, respectively). All other comparisons did not reach significance (*d*s = 0.22 to 0.24). Finally, there was a significant main effect of sex *F*(1, 927) = 5.69, *p* = .017, η_p_^2^ < 0.01, with girls having significantly higher procrastination than boys (*d* = 0.22).

**Table 2:** Descriptive statistics for procrastination scores as a function of sex and ethnicity

Sex	Ethnicity	*M*	*SD*	*n*
Boys	European	2.79	0.90	198
	Kanak	3.14	0.81	225
	Polynesian	3.36	0.75	51
Girls	European	2.93	0.86	177
	Kanak	3.33	0.81	227
	Polynesian	3.53	0.89	49
Total	European	2.86	0.88	375
	Kanak	3.24	0.82	452
	Polynesian	3.44	0.82	100

### Regression analysis

Table 3 shows the results of linear models analyses for the total sample and within ethnic group separately. For the total sample, higher procrastination scores were significantly associated with ethnicity (being Kanak or Polynesian), sex (being female), and higher age. Ethnic identity, area of residence, and SES were not significantly associated with procrastination scores in this analysis. In European adolescents, the regression was significant, higher procrastination scores being significantly associated with higher age. In Kanak adolescents, the regression was likewise significant, with higher procrastination scores significantly associated with greater ethnic identity and higher age. In Polynesian adolescents, by contrast, the regression did not reach significance.

**Table 3:** Linear regression models examining predictors of procrastination among ethnic group

*R*^2^ (Adj. *R*^2^)	Total sample (*N* = 927)			European (*N* = 452)			Kanak (*N* = 375)			Polynesian (*N* = 100)		
	.11 (0.10)			.06 (0.04)			.08 (0.06)			.10 (0.04)		
	*F*(8, 927) = 13.84 *p* < .001			* F*(6, 374) = 3.56 *p* = .002			* F(*6, 451) = 6.17 *p* < .001			* F*(6, 99) = 1.76 *p* = .116		
Variable	B	β	*p*	B	β	*p*	*B*	β	*p*	*B*	β	*p*
Melanesian^#^	0.29	0.16	< 0.001									
Polynesian^#^	0.51	0.18	< 0.001									
MEIM score	0.09	0.06	0.061	− 0.08	− 0.06	0.262	0.22	0.14	0.002	0.33	0.21	0.052
Girls^†^	0.15	0.08	0.008	0.16	0.09	0.082	0.14	0.09	0.055	0.07	0.04	0.661
Urban^$^	− 0.01	− 0.01	0.872	− 0.10	− 0.05	0.341	0.09	0.04	0.431	0.09	0.05	0.668
High SES^£^	− 0.09	− 0.05	0.240	− 0.30	− 0.16	0.076	− 0.07	− 0.04	0.469	0.00	0.00	0.999
Mid SES^£^	0.02	0.01	0.800	− 0.22	− 0.10	0.228	0.11	0.06	0.234	− 0.20	− 0.11	0.323
Age	0.13	0.18	< 0.001	0.13	0.18	< 0.001	0.11	0.17	< 0.001	0.12	0.18	0.071

Reference is: ^†^Boys; ^$^rural area; ^£^Low SES, ^#^European

**Fig. 1:**
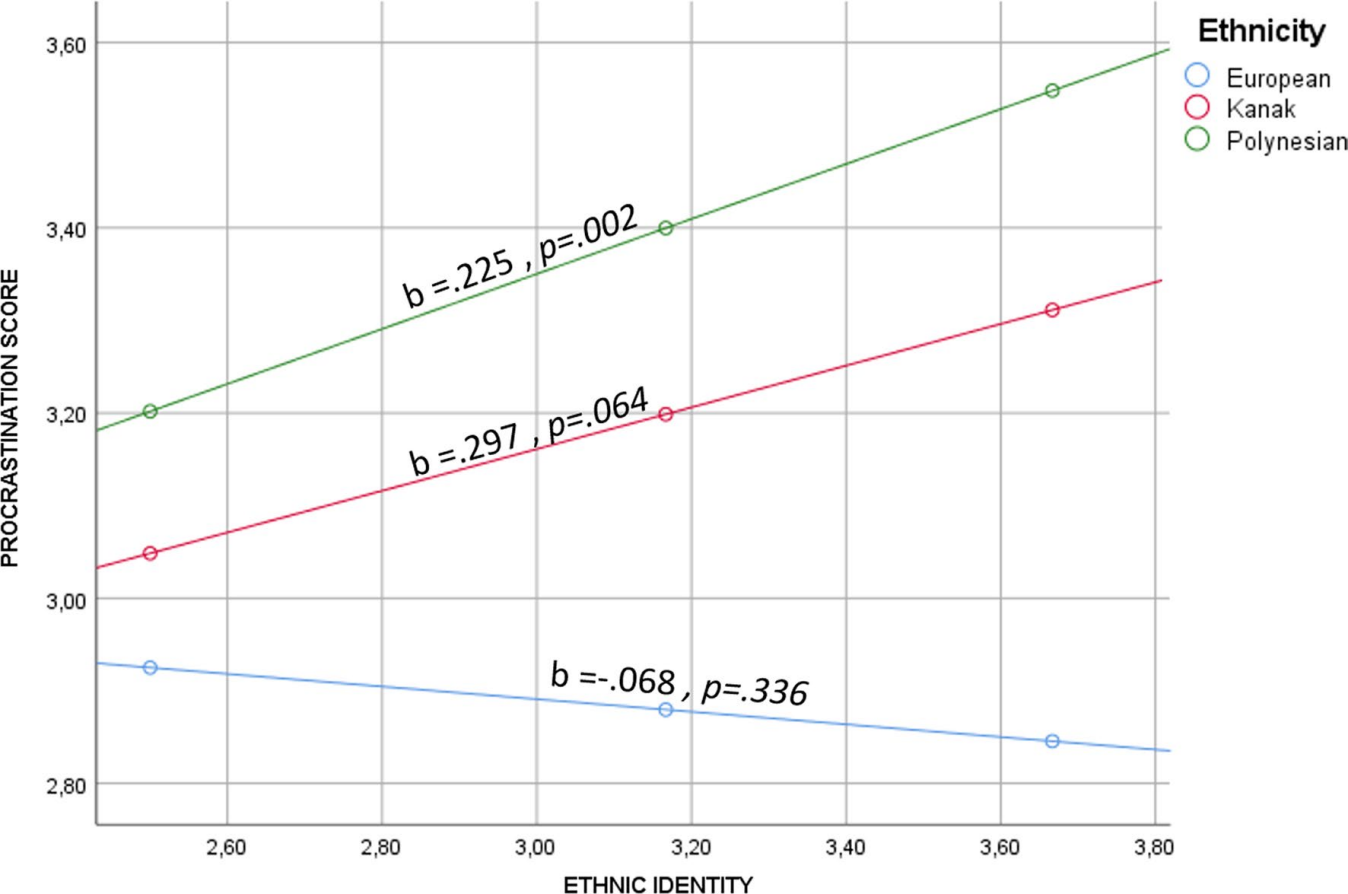
Interaction between ethnic identity and ethnicity on procrastination score

Because of the difference found within ethnic groups, notably concerning the impact of ethnic identity, we computed moderation models to better examine how ethnicity moderates the relationship between ethnic identity and procrastination. The outcome variable was procrastination scores, the moderator was ethnicity, and the predictor was MEIM scores. Other variables (SES, gender, age and area of residence) were added as covariates. After adding the interaction term (ethnicity x MEIM scores), *R*² changed significantly (Δ*R* ²= 0.010, *p* = .007). The conditional effect of ethnic identity scores was significant for Kanak adolescents (*b* = 0.225, *p* = .002), but not for European or Polynesian adolescents (*b* = − 0.068, *p* = .336, *b* = 0.297, *p* = .064, respectively), indicating higher procrastination in Kanak adolescents with higher ethnic identity scores (Fig. 1).

## Discussion

Studies of procrastination have primarily focused on adult populations and the smaller body of work on adolescent procrastination has typically relied on samples from Western, educated, industrialised, rich, and democratic nations. In the present study, we conducted an examination of adolescent procrastination in a hitherto under-researched population, namely New Caledonian adolescents. There were a number of important findings in the present study. First, we found that girls had significantly higher procrastination than boys. Second, we found significant between-group differences in procrastination as a function of ethnicity. Third, in regression analyses, we found that—beyond sex and ethnicity effects—procrastination was also significantly associated with older age and the ethnicity by ethnic identity interaction. Some of our findings stand in contrast to both our hypotheses and the findings of other studies, so we highlight these differences below.

In terms of sex differences, we found that girls had significantly higher procrastination than boys. This stands in contrast to findings among adults, where men have generally been found to have significantly higher levels of procrastination than women [31, 32]. The literature on sex differences in adolescence is more equivocal [19, 33, 34] but to our knowledge ours is the first study to report that girls have significantly higher procrastination scores than boys. One possible explanation for our finding is that the relationship between procrastination and sex is affected by national or cultural factors [32]. In the context of New Caledonia, for instance, there may be greater cultural pressure on boys to be successful and to be responsible. As a result, boys may experience greater fear of failure and consequently may be less likely to take risks on tasks [53]. Boys may also be subjected to stricter control during adolescence (e.g., being assigned more responsibility on tasks), which may mean they have fewer avenues to behaviourally express risk-taking, such as through procrastination. It should be noted, however, that the effect size of the sex difference in the present study was small (*d* = 0.22), which warrants further investigation before firm conclusions are drawn.

In terms of ethnic differences, our results indicated that Kanak and Polynesian adolescents had significantly higher levels of procrastination than European adolescents. It is difficult to explain these differences, especially given dearth of research focused on adolescent procrastination in multi-ethnic nations. One possibility is that it is reflective of ethnic differences in *time perspective*, that is, an individual’s understanding of one’s psychological past, present, and future [54]. In particular, low future time perspective has been associated with greater procrastination [54] and, importantly, non-European populations in the South Pacific and neighbouring regions have been found to have lower future time perspective than European populations [55]. Another possibility is that European adolescents in New Caledonia experience relatively greater societal pressure to attain success, and as result are less likely to procrastinate. Conversely, the more traditional lifestyles of Kanak and Polynesian adolescents may mean that they experience less strict control during adolescence and, as a consequence, have more opportunities to risk-take over tasks.

Here, we also examined the extent to which ethnic identity interacts with ethnicity, but with the exception of a significant and weak effect in Kanak adolescents, our findings in this regard were mainly null. However, in Kanak adolescents specifically, we did find that a conditional effect of ethnic identity, such that Kanak adolescents who more strongly identified with their ethnicity were more likely to have higher procrastination scores. This broadly supports our contention that ethnic identity (beyond ethnicity *per se*) complicates how procrastination manifests in specific cultural contexts. Indeed, previous work in the New Caledonian context has shown that greater ethnic identity was associated with significantly higher self-esteem [42]. However, explaining why ethnic identity moderated the relationship between ethnicity and procrastination only in Kanak adolescents is more difficult, particularly as we expected similar relationships across ethnic groups. This is certainly an area that is worthy of further investigation.

In terms of age, we found a positive relationship between age and procrastination scores in our sample. In fact, of the variables included in our analyses, age was the strongest correlate of procrastination. This was contrary to our expectation of a negative relationship [16] and requires some consideration. One possibility is that, within this age group (i.e., 10- to 16-year-olds), levels of procrastination are impacted by the changes in the school system. That is, as adolescents advance through the schooling system, they may begin to procrastinate more when faced with more anxiety-inducing tasks [20, 21]. Relatedly, older adolescents may also be given greater responsibilities or given individual, rather than group-based tasks, which may lead to increased procrastination. An alternative possibility is that our findings are reflective of developmental changes in Conscientiousness [56], a trait that is essential for task completion and responsibility. Although Conscientiousness generally increases with advancing age, it may be that developmental trajectories remain unstable in adolescence and, consequently, have an impact on levels of procrastination. It is important to note, however, that we are not aware of any previous studies that have examined developmental trajectories of procrastination in adolescence and, given our findings, this seems to be worthy of further investigation.

A strength of the present work is the focus on procrastination in an adolescent population that has generally been under-researched. Nevertheless, a number of limitations of the present study should be considered. First, it is unlikely that our sample is representative of the wider adolescent population in New Caledonia. As such, caution should be exercised when thinking about generalising our findings more broadly, whether in New Caledonia or in other Pacific populations. Second, our subsample sizes may have been too small in some cases. We conducted a *post hoc* power calculation, which indicated that our sample size was adequately powered for regression analysis (power = 0.93), but was slightly less-than-adequate for between-group analyses in terms of ethnicity (power = 0.89). As such, it may be important to replicate our findings with larger groups of New Caledonian adolescents. While data for this study was collected prior to the COVID-19 pandemic, the literature showed that procrastination may have been negatively impacted by COVID-19-related factors [57, 58] and, as such, it would be interesting to replicate our work to determine whether any change has occurred in the New Caledonian context. Finally, we remind readers that this is a preliminary study: there is much more that could be done to better understand procrastination in this population. This might include a focus on parental control and attachment styles [26, 27], as well as outcomes of adolescent procrastination, such as academic performance and psychological well-being [2, 7].

## Conclusion

These limitations notwithstanding, our work contributes to the literature on adolescent procrastination by considering the case of a hitherto under-researched population. Our findings are noteworthy, as they suggest that there are moderate-to-large ethnic differences and smaller sex differences in procrastination among New Caledonian adolescents. These results may have important implications for educational programs in New Caledonia. For instance, to the extent that procrastination is associated with poorer academic performance [7], ethnic and sex differences in procrastination may have downstream differential impacts on school achievement. Practitioners working with adolescents may find it useful to consider these differences in their everyday practice and tailor their pedagogic methods appropriately, so that no one group is negatively impacted [53]. From a scholarly point-of-view, further research on procrastination in adolescent populations in samples that have traditionally featured in the research literature is urgently needed.

## Data Availability

The datasets used and analysed during the current study are available from the corresponding author on reasonable request.
